# SABCS 2017: update on chemotherapy, targeted therapy, and immunotherapy

**DOI:** 10.1007/s12254-018-0430-0

**Published:** 2018-08-17

**Authors:** Rupert Bartsch, Elisabeth Bergen

**Affiliations:** Department of Medicine 1, Clinical Division of Oncology, Comprehensive Cancer Centre Vienna, Medical University of Vienna, Waehringer Guertel 18–20, 1090 Vienna, Austria

**Keywords:** Breast cancer chemotherapy, HER2-positive disease, Highlights, San Antonio Breast Cancer Symposium 2017, Triple-negative disease

## Abstract

In the areas of chemotherapy, targeted therapy and immunotherapy, several interesting and clinically relevant data were presented at the 2017 San Antonio Breast Cancer Symposium (SABCS). This short review focuses on dose-dense and/or sequential administration of adjuvant chemotherapy, provides an update on targeted therapies for HER2-positive and triple-negative breast cancer and summarizes new results in the field of immunotherapy.

## Introduction

Results of several clinically relevant studies were presented at the 2017 San Antonio Breast Cancer Symposium (SABCS). This short review provides an update on novel data in the fields of chemotherapy, targeted therapy and immunotherapy.

## Dose-dense and/or sequential chemotherapy

The term dose-dense chemotherapy refers to the application of chemotherapeutic drugs at standard dose in shorter intervals (i.e., every two weeks instead of once every three weeks). Based upon a Gompertzian model of tumour-growth, dose-dense strategies increase the log-kill of tumour cells, thereby effectively preventing cancer regrowth [1]. Sequential as opposed to concurrent administration of chemotherapy is another attractive way of increasing the activity of adjuvant treatment; this approach allows for the administration of each drug at the maximum tolerated dose [2]. In order to further elucidate the role of dose-dense and sequential chemotherapy, the Early Breast Cancer Trialists’ Collaborative Group (EBCTCG) conducted a meta-analysis of prospective randomised phase III trials comparing dose-dense and/or sequential regimens with conventional adjuvant chemotherapy.

In total, individual data of 34,122 patients included into 25 trials were available [3]. Dose-dense and/or sequential administration of chemotherapy resulted in a consistent reduction of recurrence risk and breast cancer-specific mortality. Furthermore, a pooled analysis including all 25 studies reported a similar reduction of all-cause mortality (RR [relative risk] 0.87; 95% CI 0.82–0.91; 10-year gain 3.0%).

In summary, these data indicate a relevant benefit for dose-dense and/or sequential chemotherapy regimens compared with conventionally dosed treatment in terms of recurrence risk and mortality; superiority was seen in all subgroups and independent of hormone-receptor status. In addition, no increased risk of alternative mortality (due to increased toxicity of more intense adjuvant treatment) was observed as shown by the sustained effect on all-cause mortality and no increase of rates of death without recurrence. Despite these clear-cut data, it needs to be stressed that the use of three-weekly paclitaxel in the control groups of several studies investigating dose-dense regimens may have influenced this outcome as it is well known that three-weekly paclitaxel is a suboptimal way of taxane administration. In addition, no advantage of dose-dense administration was shown for docetaxel to date [4]. Therefore, dose-dense and/or sequential chemotherapy is a potential adjuvant treatment standard but should not be regarded as the only appropriate way to administer adjuvant chemotherapy in early stage breast cancer.

## GeparSepto

The neoadjuvant German Breast Group (GBG) GeparSepto trial investigated the substitution of solvent-based paclitaxel by nanoparticle albumin-bound (nab) paclitaxel as a component of neoadjuvant treatment. As already published, nab-paclitaxel increased pathologic complete response (pCR) rates in the overall population with the largest benefit observed in triple-negative breast cancer (TNBC; [5]). Of note, pCR predicts improved long-term outcome on an individual patient level in high-risk breast cancer subtypes; still, a correlation of pCR with long-term outcome has not yet been convincingly demonstrated on a trial level [6]. At the 2017 SABCS, disease-free survival (DFS) data of GeparSepto were presented [7]. At a median follow-up of 49 months, nab-paclitaxel significantly reduced breast cancer recurrence risk by an absolute 6.4% (HR [hazard ratio] 0.69; 0.54–0.89). This effect was observed in all of the predefined subgroups and surprisingly most pronounced in tumours with low proliferation rate. The DFS associated with nab-paclitaxel was in the range expected from the pCR increase in TNBC (and the results therefore indicate a correlation of pCR and DFS) while a residual effect beyond pCR must be assumed in luminal breast cancer where only a small pCR delta was observed. In addition, these data suggest that luminal breast cancer patients should not be excluded from neoadjuvant chemotherapy studies *per se*.

## HER2-positive breast cancer

The phase III SOLD study was the fourth trial to compare short-term trastuzumab administration with the current standard of one year of adjuvant treatment [8]. The intent-to-treat population consisted of 2174 patients; at a median follow-up of 5.2 years, non-inferiority of 9 weeks of weekly trastuzumab could not be established (DFS HR 1.39; 95% CI 1.12–1.72). These results again indicate that one year of adjuvant immunotherapy remains the standard of care.

The German GAIN-2 trial included a substudy conducted in HER2-positive patients comparing trastuzumab bioavailability of two different places of subcutaneous application [9]. Administration into the thigh according to standard practice resulted in a 30% higher bioavailability of trastuzumab as compared to administration into the abdominal wall. While no cross-over design was included and the clinical relevance of these findings remain unclear, results from the GAIN-2 HER2-substudy indicate that the thigh remains the preferred place of subcutaneous trastuzumab administration.

A central review of the HER2-status in North American adjuvant trastuzumab studies suggested that a benefit for trastuzumab may exist in patients with HER2 low-expressing tumours [10]. This led to the phase III NSABP B‑47 trial comparing an additional one-year of adjuvant trastuzumab after standard chemotherapy with no immunotherapy in patients with HER2 1+ or 2+ (ISH negative) breast cancer [11]. Disappointingly, no difference between the two arms was observed (HR 0.98; 95% CI 0.77–1.26) indicating that trastuzumab should be reserved for HER2-positive tumours as defined by HER2 overexpression and/or *HER2/neu* gene amplification.

The phase Ib/II PANACEA trial (KEYNOTE-014) evaluated the combination of trastuzumab with the immune-checkpoint inhibitor pembrolizumab in metastatic breast cancer patients progressing on prior HER2-directed therapy [12]. The study was based upon preclinical data suggesting that this combination may offer the chance to overcome trastuzumab resistance [13]; safety and efficacy of trastuzumab plus pembrolizumab in HER2-positive metastatic breast cancer patients with PD-L1 expressing tumours was defined as the primary study endpoint. A second, smaller, PD-L1 negative cohort was included as well. Overall, 58 patients were accrued; all had received prior trastuzumab-containing therapy and 51 additional anti-HER2 therapy as well (including pertuzumab and T‑DM1). Overall response rate (ORR) in the PD-L1 positive cohort was 15% (90% CI 7–29) and an encouraging disease-control rate (DCR; CR + PR + SD ≥ 6 months) of 25% (90% CI 14–49) was reported, while no relevant activity was seen in PD-L1 negative subjects. Median progression-free survival (PFS) in the PD-L1 positive cohort was only 2.7 months but patients responding to treatment experienced prolonged disease control.

## Update immunotherapy

Besides PANACEA, several other studies on the potential role of immunotherapy in breast cancer were presented. In the phase II trial KEYNOTE-086, patients with metastatic TNBC received single-agent pembrolizumab [14]. Two different patient cohorts were accrued. As already reported, activity of pembrolizumab in pretreated patients (cohort A; *n* = 170) was low with a response rate of 4.7%; in the first-line cohort, however (cohort B; *n* = 84), an encouraging response rate of 23% (95% CI 15–33%) was observed indicating relevant clinical activity.

Several combination trials of checkpoint inhibitors with other agents such as eribulin [15], abemaciclib [16], or olaparib [17] were presented as well. All of these trials suggested clinical activity and treatment was generally well tolerated with no overlapping toxicity observed. These studies, however, were limited by their non-randomized designs and the fact that activity was within the range expected from single-agent treatment. Therefore, randomized trials are required in order to fully assess the clinical potential of these strategies in metastatic breast cancer.

## Sacituzumab govitecan (IMMU-132)

IMMU-132 is regarded as being one of the most promising agents for the treatment of TNBC. This antibody-drug conjugate (ADC) consists of a humanized antibody targeting Trop-2 (tumour-associated calcium signal transducer 2) linked to SN-38, the active metabolite of irinotecan [18]. Of note, Trop-2 is commonly overexpressed in epithelial cancers and >90% of TNBC have high Trop-2 expression.

In total, 110 patients with metastatic TNBC and ≥2 treatment lines were accrued to a single-arm phase II trial [19]. Confirmed ORR was 34% and DCR 46%; median PFS was 5.5 months. Treatment was overall well tolerated with the majority of ≥3 grade adverse events consisting of neutropenia (39%), leukopenia (14%), and anaemia (10%). In summary, these data suggest superior activity of IMMU-132 compared with conventional chemotherapy in this setting.

## EMBARCA

The OlympiaD trial established that the PARP-inhibitor olaparib was superior to treatment by physician’s choice (TPC) in metastatic breast cancer patients harbouring germ-line (g) *BRCA* mutations [20]. The randomized phase III EMBARCA trial compared the novel PARP inhibitor talazoparib with TPC (capecitabine, eribulin, gemcitabine, vinorelbine) in 431 pretreated or previously untreated patients with metastatic breast cancer and *gBRCA* mutations [21]. Median PFS in the talazoparib group was 8.6 months as compared with 5.6 months in the TPC arm (HR 0.54; 95% CI 0.41–0.71; *p* < 0.0001). This benefit was observed in all predefined subgroups and even patients with brain metastases upon inclusion benefitted from PARP-inhibitor therapy. Taken together, data from EMRACA and OlympiAD suggests that PARP inhibitors when available in the clinical routine setting will have an important role in this patient subset.

## Summary

Several clinically relevant studies were presented at the 2017 SABCS. Dose-dense and/or sequential administration of chemotherapy is a potential standard in the adjuvant treatment of early stage breast cancer patients. Immunotherapy, PARP inhibitors or ADCs such as sacituzumab govitecan may ultimately change the treatment strategies in metastatic breast cancer.
